# Disease Progression in Iatrogenic Cerebral Amyloid Angiopathy

**DOI:** 10.1212/WNL.0000000000218511

**Published:** 2026-09-29

**Authors:** Simon Fandler-Höfler, Benedetta Storti, Kanishk Kaushik, Larysa Panteleienko, Slaven Pikija, Fabio Rossini, Payam Tabaee Damavandi, Isabella Canavero, Giulia Negro, Jacopo C. DiFrancesco, Christian Enzinger, Thomas Gattringer, Ellis S. Van Etten, Anna Bersano, Marieke J.H. Wermer, Gargi Banerjee, David John Werring

**Affiliations:** 1 Department of Neurology, Medical University of Graz, Austria;; 2 Stroke Research Centre, Department of Translational Neuroscience and Stroke, UCL Queen Square Institute of Neurology, United Kingdom;; 3 Cerebrovascular Unit, Fondazione IRCCS Istituto Neurologico Carlo Besta, Milan, Italy;; 4 Department of Neurology, Leiden University Medical Center, Leiden, the Netherlands;; 5 Department of Neurology, Bogomolets National Medical University, Kyiv, Ukraine;; 6 Department of Neurology, Christian-Doppler-Clinic, Paracelsus Medical University, Salzburg, Austria;; 7 Department of Neurology, Fondazione IRCCS San Gerardo dei Tintori, Monza, Italy;; 8 Department of Neurology, University Medical Center Groningen, University of Groningen, the Netherlands; and; 9 MRC Prion Unit at UCL, Institute of Prion Diseases, London, United Kingdom.

## Abstract

**Background and Objectives:**

Iatrogenic cerebral amyloid angiopathy (iCAA), likely caused by transmission of amyloid-beta by medical procedures, is a rare but distinct cerebral amyloid angiopathy (CAA) subtype, with rising numbers of patients identified globally. However, given its relatively recent recognition, data on long-term outcomes and prognosis are scarce. Therefore, we aimed to investigate the clinical disease progression in patients diagnosed with iCAA.

**Methods:**

After a systematic literature search for larger case series of iCAA (n ≥ 5), we included patients from 4 European countries (Austria, Italy, The Netherlands, and the United Kingdom) meeting modified Queen Square criteria for possible or probable iCAA. Clinical outcomes of interest were symptomatic intracerebral hemorrhage (ICH), convexity subarachnoid hemorrhage (cSAH), cognitive status at last follow-up, and mortality. We used Cox regression to investigate clinical variables associated with those outcome events.

**Results:**

We included 82 patients (59.8% male, median age at presentation 50 years, median latency from exposure 39 years); 50% of patients initially presented with ICH and 32.9% with cSAH/transient focal neurologic episodes. During a median follow-up time of 3.8 years (IQR 1.8–5.9; total follow-up 385 patient-years), 74 symptomatic ICH occurred, corresponding to a rate of 19.2 per 100 patient-years after first presentation. Patients who initially presented with ICH had an increased rate of recurrent ICH compared with other presentations (hazard ratio 3.14, 95% CI 1.55–6.33, *p* = 0.001) with a recurrence rate of 24.6 per 100 patient-years (95% CI 18.2–32.6). Patients with spinal surgery as an exposure (n = 24) had a lower rate of ICH compared with brain surgery or embolization (hazard ratio 0.34, 95% CI 0.13–0.87, *p* = 0.02). Six patients (7.3%) died during the follow-up period (initial presentation with ICH in 3, cSAH in 3). Cognitive impairment at last available cognitive evaluation (median 1.9 years from first presentation) was recorded in 42 patients (51.2%).

**Discussion:**

Following a long latency from presumed exposure, patients diagnosed with iCAA have a high rate of subsequent symptomatic ICH (∼20% yearly risk), with recurrence risk being the highest among patients initially presenting with ICH and those exposed by brain rather than spinal surgery. The mortality rate was low despite the high frequency of ICH.

## Introduction

Iatrogenic cerebral amyloid angiopathy (iCAA) is a rare, but increasingly recognized, cerebral amyloid angiopathy (CAA) subtype likely caused by the transmission of amyloid-ß pathology by medical procedures, particularly neurosurgery involving cadaveric dural material.^1,2^ After initial clinical reports in 2019,^3^ cohorts of patients with iCAA have been identified,^4-6^ with diagnostic criteria proposed^1^ and updated.^6^ Given that cadaveric dural grafts such as Lyodura were widely used between the 1970s and early 1990s and that the median latency period for clinical presentation of iatrogenic CAA is approximately 35–40 years,^6^ continued identification of newly symptomatic patients in the coming years is likely.

Most patients with iCAA are identified after clinical presentation with intracerebral hemorrhage (ICH) or convexity subarachnoid hemorrhage (cSAH).^4-6^ In recent years, advances have been made in defining the clinical and neuroradiologic phenotypes,^6,7^ susceptibility,^8^ biomarker profiles,^9^ and affected age groups.^10^ However, evidence on longer-term outcomes (of major importance to both patients and health care professionals) remains limited, because of the limited sample size and insufficient follow-up duration of individual cohorts, with smaller case series potentially susceptible to ascertainment bias.

With data on the growing number of patients with increasing follow-up time available, we therefore aimed to address this knowledge gap by investigating clinical outcomes (occurrence of ICH and cSAH, cognitive status and mortality) in an international multicenter cohort study.

## Methods

We pooled individual patient data from previously published iCAA cohorts. We once again approached centers that had contributed to a recent collaborative effort investigating MRI characteristics of iCAA, identified following a systematic search of case series including ≥5 patients with iCAA,^6^ and invited those to participate in this multicenter study. Although our previous study focused on MRI characteristics, this study aims to assess clinical outcomes over longer follow-up periods in an extended study cohort. In addition to previously published cases, we also included patients with iCAA subsequently identified by the participating centers.

To be included in this study, patients needed to meet the modified Queen Square criteria for probable or possible iCAA^6^; we also required all patients to have at least 2 hemorrhagic lesions for a diagnosis of iCAA (Figure 1). This change did not result in any patient exclusions.

**Figure 1:**
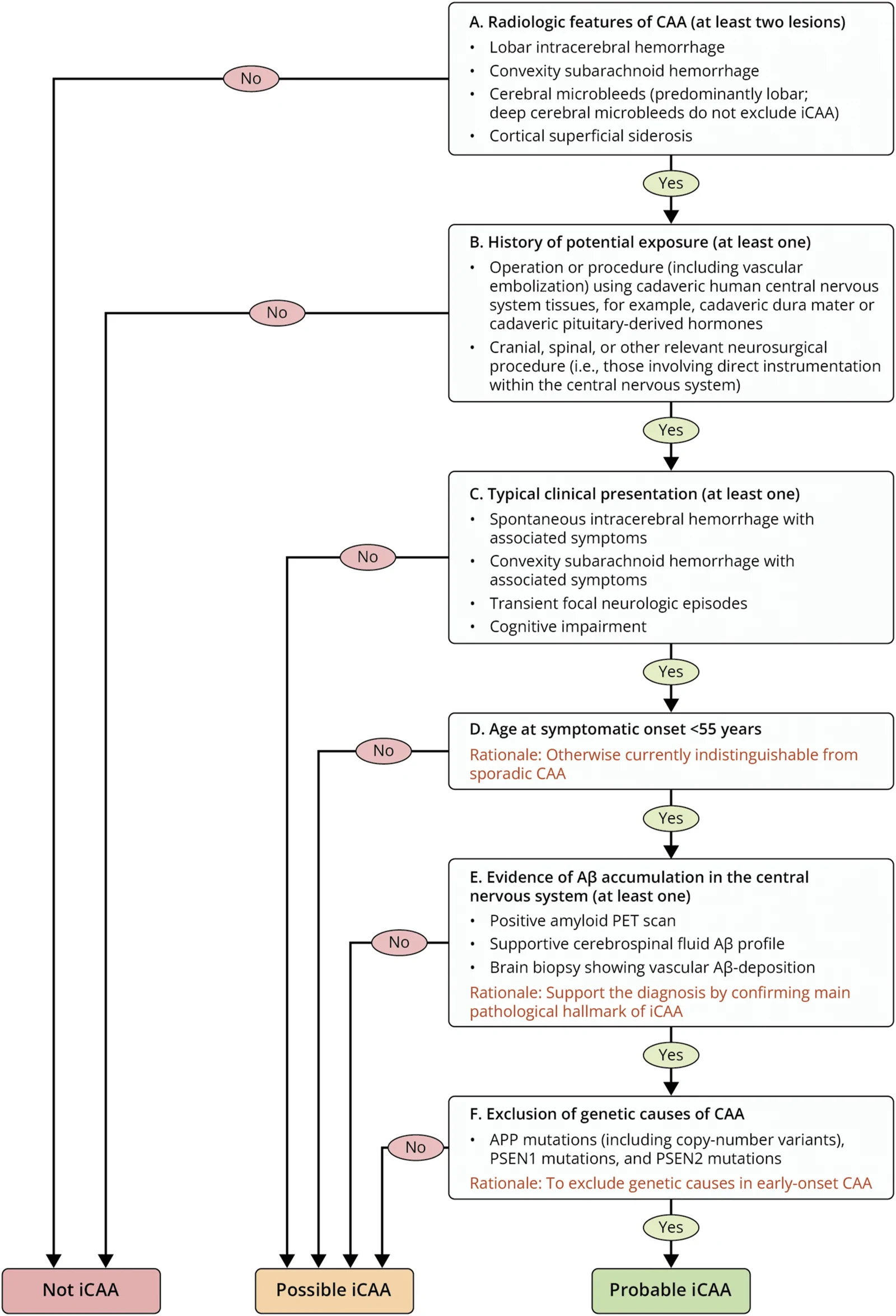
Flowchart for Using the Modified Queen Square Criteria for Iatrogenic Cerebral Amyloid Angiopathy^6^ CAA = cerebral amyloid angiopathy; iCAA = iatrogenic cerebral amyloid angiopathy.

Data were collected at each center using a standardized case report form, capturing demographic variables, details of presumed exposure, clinical presentation, diagnostic workup, and longitudinal follow-up. Follow-up schedules generally consisted of annual outpatient clinical and neuroradiologic assessments in all centers but varied in individual patients based on their clinical needs. Outcome events were assessed at the treating center and included the occurrence (or recurrence) of ICH, cSAH, changes in cognitive status, and mortality.

Cognitive examinations at baseline and follow-up varied but usually consisted of a full neuropsychological assessment or included at least the Montreal Cognitive Assessment (MoCA). Cognitive impairment was defined as either: MoCA <26/30, Mini-Mental State Exam <24/30, scores <1.5 standard deviations below normative means in ≥1 domain in multidomain neuropsychological assessments, or a clinical diagnosis of dementia.

### Statistical Analysis

We performed statistical analysis using IBM SPSS Statistics for Windows, version 30 (IBM Corp, Armonk, USA). We used descriptive statistics to present the main findings and calculated incidence rates of ICH and cSAH. Differences between exposure groups (supratentorial surgery, infratentorial surgery, spinal surgery, embolizations) were assessed according to baseline variables using Chi-square tests for categorical variables and *t* tests or nonparametric tests for continuous variables, as appropriate. Cox regression was used to estimate hazard ratios of factors potentially associated with the risk of (recurrent) ICH during the follow-up period, including exposure groups and types of symptomatic presentation.

### Standard Protocol Approvals, Registrations, and Patient Consents

The study was approved by the ethics committee of the Medical University of Graz. Included patients either gave written informed consent or had consent waived for the use of retrospective data based on local ethics committee approvals at participating centers.

### Data Availability

The data sets generated during this study are available from the corresponding author upon reasonable request.

## Results

### Study Cohort Characteristics

We included 82 patients (59.8% male, Table 1). The median age at exposure was 8 years (interquartile range [IQR] 3–20 years). The exposure events were supratentorial brain surgery in 44 patients (53.7%), infratentorial brain surgery in 10 patients (12.2%), spinal surgery in 24 (29.2%), and embolization through head or neck vessels in 4 patients (4.9%). The underlying indications for these procedures included neoplasm (37.8%), traumatic brain injury (31.7%), congenital anomalies (20.7%), and other causes (9.8%). Thirty-one patients (37.8%) met criteria for probable iCAA and 51 patients (62.2%) met criteria for possible iCAA.

**Table 1:** Clinical Characteristics of the Study Cohort (n = 82)

Demographical data	
Male sex (n, %)	49 (59.8)
Age at exposure in y (median, IQR)	8 (3–20)
Country	
Italy (n, %)	33 (40.2)
The Netherlands (n, %)	25 (30.5)
The United Kingdom (n, %)	17 (20.7)
Austria (n, %)	7 (8.5)
Exposure event type	
Supratentorial brain surgery (n, %)	54 (53.7)
Infratentorial brain surgery (n, %)	10 (12.2)
Spinal surgery (n, %)	24 (29.2)
Head and neck embolization (n, %)	4 (4.9)
Symptomatic presentation	
Intracerebral hemorrhage (n, %)	41 (50.0)
Convexity subarachnoid hemorrhage/transient focal neurologic episode (n, %)	27 (32.9)
Seizures (n, %)	6 (7.3)
Cognitive decline (n, %)	3 (3.7)
Incidental diagnosis (n, %)	5 (6.1)
Latency from exposure, time in y (median, IQR)	39 (35–42)
Age at symptom onset/diagnosis, y (median, IQR)	50 (43–60)
Follow-up events	
Follow-up duration in y (median, IQR)	3.8 (1.8–5.9)
Any intracerebral hemorrhage (n, %)	41 (50.0)
Any convexity subarachnoid hemorrhage (n, %)	10 (12.2)
Mortality (n, %)	6 (7.3)
Cognitive impairment (n, %, missing in 5 patients)	36 (48.8)

### Clinical Presentation

The median age at presentation was 50 years (IQR 43–60), and the median latency between exposure event to first clinical presentation was 39 years (IQR 35–42).

The most frequent presenting event was spontaneous ICH (n = 41, 50%), followed by cSAH, transient focal neurologic episodes, or both (32.9%), seizures (7.3%), and cognitive decline (3.7%). In addition, 5 patients (6.1%) were found to have CAA incidentally on neuroimaging performed for other, likely unrelated, indications (headache, n = 3; routine surveillance for a neurosurgical shunt, n = 1; no recorded indication, n = 1).

### Progression During Follow-Up

The median follow-up time was 3.8 years (IQR 1.8–5.9) with a total follow-up duration of 385 patient-years. Twelve patients (14.6%) were lost to follow-up. Follow-up assessments were mostly performed in-person (74.4%), but also through telephone (25.6%). In addition to the 41 ICH index events, 74 incident recurrent ICH occurred during the follow-up period in 41 patients (28 of whom had an ICH as index event and 13 did not). In the overall cohort, the rate of incident ICH during follow-up after iCAA diagnosis was 19.2 per 100 patient-years (95% CI 15.5–23.9) overall and 24.6 per 100 patient-years in patients who presented with ICH (95% CI 18.2–32.6). We observed 12 incident cSAH (in 10 patients) during the follow-up period (3.1 per 100 patient-years, 95% CI 1.6–5.5). Six patients (7.3%) died during the follow-up period (3 due to recurrent ICH, 3 due to unknown causes, Figure 2). Of those 6 patients, 3 initially presented with ICH and 3 with cSAH.

**Figure 2:**
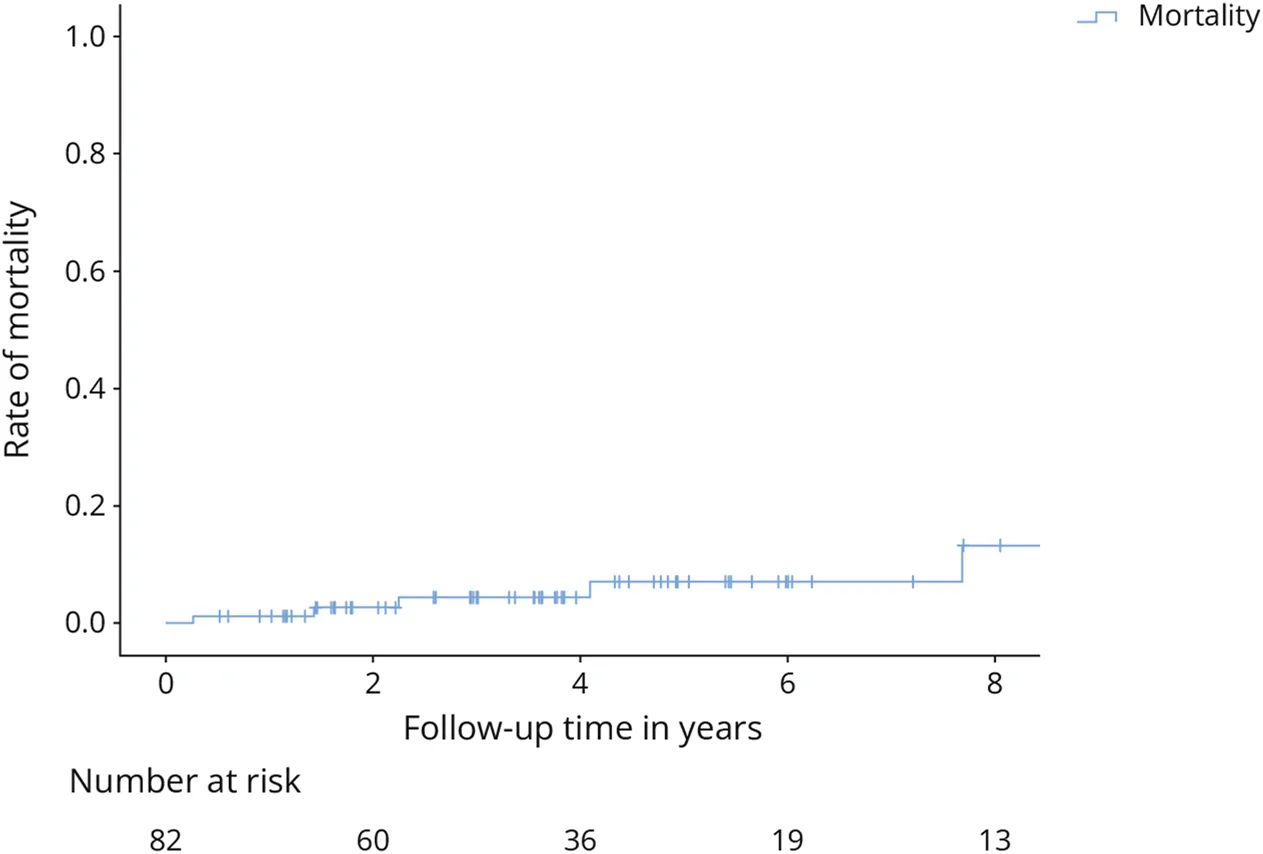
Rate of Mortality of Patients With Iatrogenic Cerebral Amyloid Angiopathy

Regarding cognitive outcomes, 34 patients (41.5%) underwent a full neuropsychological assessment, 23 patients (28.0%) were tested using MoCA, 6 (7.3%) using the Mini-Mental State Examination, and 5 (6.1%) based on clinical history/diagnosis of dementia; 14 patients (17.1%) did not undergo any formal evaluation. Cognitive impairment at last available cognitive evaluation (median 1.9 years after clinical presentation) was present in 42 patients (51.2%).

### Factors Associated With Recurrent ICH

Age and sex were not associated with recurrent ICH events (Table 2). Patients with spinal surgery had an ICH recurrence rate of 5.4 per 100 patient-years (CI 2.1–11.4), a lower risk than those with brain surgery or embolization (hazard ratio 0.34, 95% CI 0.13–0.88, *p* = 0.02, Figure 3). When comparing patients with spinal surgery with those who had different surgical procedures (supratentorial, infratentorial, embolization), there was no difference in latency (i.e., time between exposure and clinical presentation; median 40 vs 39 years, *p* = 0.65), duration of available follow-up (median 3.6 vs 3.9 years, *p* = 0.68), rate of confirmed dural patch usage (58.3% vs 56.9%, *p* = 0.91), or rate of probable iCAA diagnosis (25.0% vs 43.1%, *p* = 0.12). No difference in ICH recurrence was observed when comparing patients with infratentorial and supratentorial brain surgery (hazard ratio 0.81, 95% CI 0.28–2.32).

**Table 2:** Characteristics of the Study Cohort According to Occurrence/Recurrence of ICH During the Follow-Up Period

	ICH during follow-up (n = 41)	No ICH (n = 41)	*p* Value
Clinical data			
Male sex (n, %)	28 (68.3)	21 (51.2)	0.64
Age at exposure in y (median, IQR)	5 (2–15)	11 (3–24)	0.39
Probable iCAA diagnosis (n, %)	20 (48.8)	11 (26.8)	0.70
Possible iCAA diagnosis (n, %)	21 (51.2)	30 (73.2)	0.70
Exposure event type			0.02
Supratentorial brain surgery (n, %)	30 (73.2)	14 (34.1)	
Infratentorial brain surgery (n, %)	4 (9.8)	6 (14.6)	
Spinal surgery (n, %)	5 (12.2)	19 (46.3)	
Head and neck embolization (n, %)	2 (4.9)	2 (4.9)	
Symptomatic presentation			0.004
Intracerebral hemorrhage (n, %)	28 (68.3)	13 (31.7)	
Convexity subarachnoid hemorrhage/transient focal neurologic episode (n, %)	8 (19.5)	19 (46.3)	
Seizures (n, %)	4 (9.8)	2 (4.9)	
Cognitive decline (n, %)	1 (2.4)	2 (4.9)	
Incidental diagnosis (n, %)	0	5 (12.2)	
Latency from exposure, time in y (median, IQR)	39 (34–42)	39 (35–44)	0.24
Age at symptom onset/diagnosis, y (median, IQR)	48 (41–57)	52 (45–63)	0.97

Abbreviations: iCAA = iatrogenic cerebral amyloid angiopathy; ICH = intracerebral hemorrhage.

**Figure 3:**
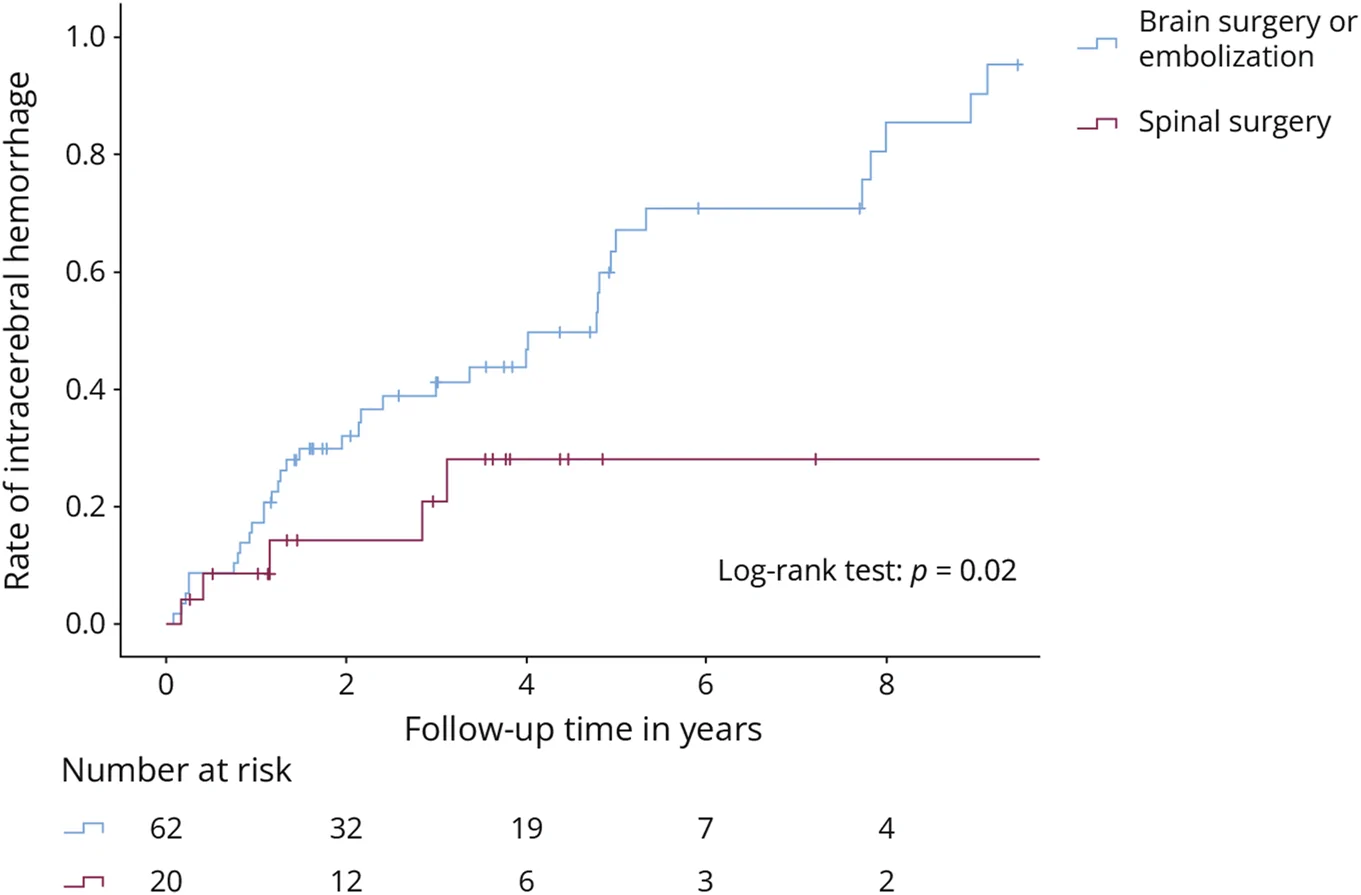
Risk of Symptomatic Intracerebral Hemorrhage in Patients Based on Type of Exposure Event

Patients initially presenting with an ICH had higher risks of (recurrent) ICH at follow-up than patients with other index presentations (hazard ratio 3.14, 95% CI 1.55–6.33, *p* = 0.001, Figure 4). No ICH events were recorded in the 5 patients incidentally diagnosed with iCAA (total follow-up time: 25 patient-years). No difference was found in the risk of incident ICH between patients with probable vs possible iCAA (*p* = 0.70, Table 2).

**Figure 4:**
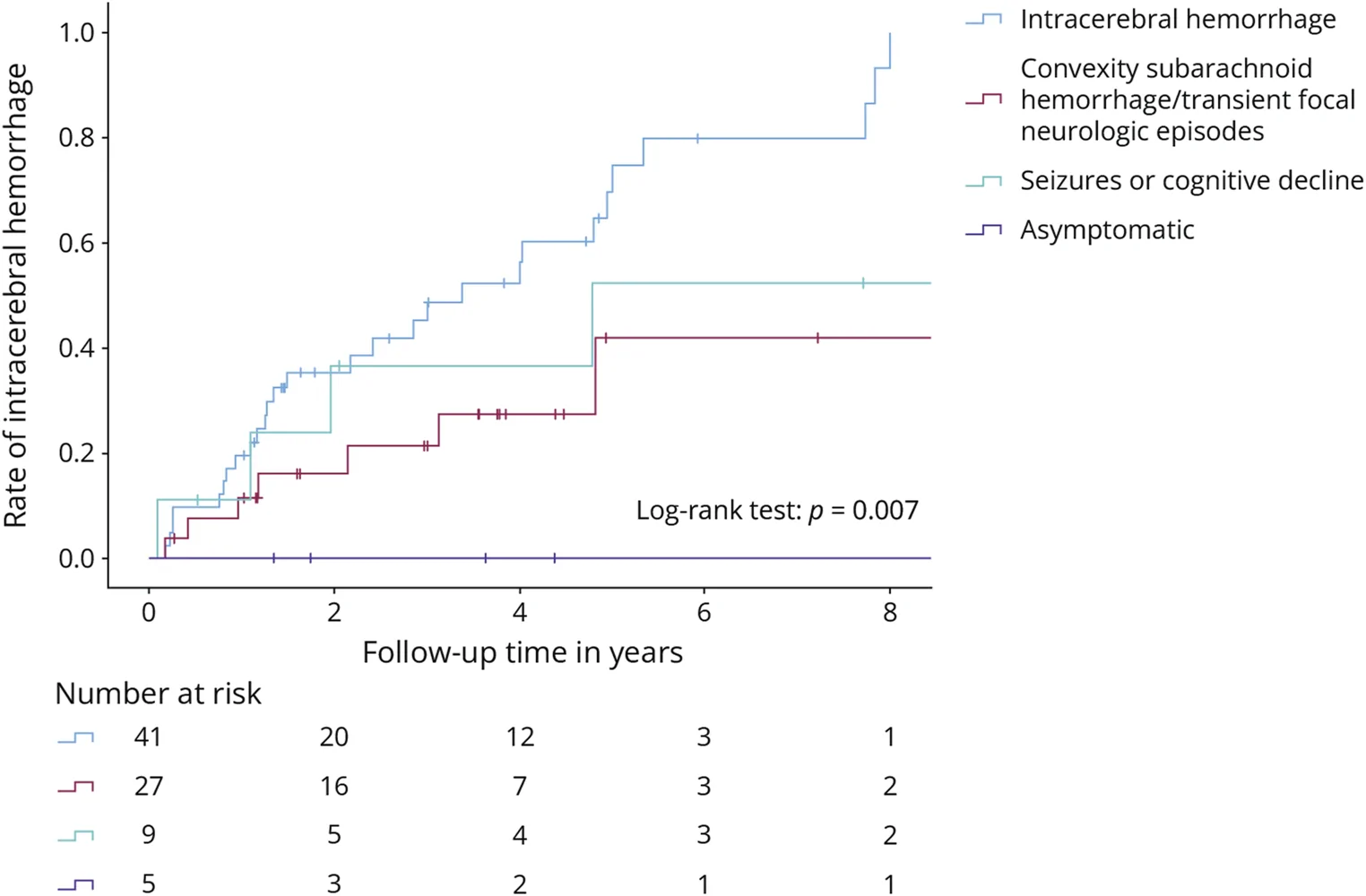
Risk of Symptomatic Intracerebral Hemorrhage in Patients According to Type of Index Presentation

## Discussion

This cohort study of the natural history of iCAA included a larger number of patients over a longer duration of follow-up compared with previous reports. We observed a very high rate of ICH in patients following diagnosis with symptomatic iCAA; following a long asymptomatic latent period of 35–45 years, patients in the symptomatic phase experienced an approximately 20% annual risk of ICH during a median 3.8 years of follow-up. This is 2 or 3 times greater than that observed in patients with ICH caused by sporadic CAA.^11,12^ Interestingly, the 5 patients who were asymptomatic at the time of diagnosis were among the minority of patients without ICH during the follow-up period. We hypothesize that these patients remain in a presymptomatic subclinical phase of iCAA; in contrast, the very high rate of ICH once the iCAA has become symptomatic is striking and suggests a rapid acceleration in disease activity from this point. These observations support the hypothesis that the risk of symptomatic hemorrhage in CAA is not uniform over time but might cluster or escalate abruptly within specific high-risk phases of the disease, as suggested by previous studies on iCAA,^6^ Dutch-type hereditary CAA,^13^ and sporadic CAA.^14,15^

Presentation with an ICH was associated with the highest risk of subsequent ICH, suggesting more advanced CAA and consistent with previous research in ICH and CAA cohorts, which showed that a history of or presentation with ICH is one of the strongest predictors of recurrent ICH in patients with CAA.^16-18^ Interestingly, despite the high rate of ICH events, the mortality rate remained relatively low. Nontraumatic ICH of any cause has a one-month mortality rate of up to 40%,^19^ and a one-year survival rate of 46%.^20^ In contrast, mortality in our cohort was substantially lower, with only 6 deaths among 56 patients who experienced 115 ICH. Potential reasons for this include: first, that patients with iCAA are younger than typical ICH populations and therefore have fewer comorbidities, which could improve survival. Second, it is possible that hemorrhages occurring in iCAA may have characteristics that are associated with better outcomes, such as smaller size and cortical or intragyral location; this interpretation is supported by our previous observation that small-volume intragyral hemorrhages are frequent in iCAA.^6^ Finally, the most severe hemorrhagic manifestations of iCAA could lead to death before the diagnosis can be considered or confirmed, resulting in a survivorship bias.

Although the risk of recurrent ICH was high for most patients, those who had spinal surgery as their exposure event (compared with other exposures) had approximately one-third the rate of recurrence. This, together with previous observations that hemorrhagic lesions in iCAA following supratentorial surgery tend to occur in the same hemisphere as the operation site,^6,21^ seem to suggest that the mode of exposure, and its anatomic proximity to the brain, or the route by which amyloid-beta seeds reach the cerebral vasculature, may have relevance to subsequent hemorrhage risk. In some ways, this is puzzling; if we consider conventional prions, experimental models of their kinetics suggest that there are 2 distinct stages: initially there is exponential prion propagation, which is clinically silent (i.e., a latent incubation period), followed by a second stage in which prion titer has plateaued but toxicity occurs, with relatively rapid onset of clinical symptoms.^22,23^ There is evidence from experimental models to suggest that amyloid-β seeds may also have two-stage kinetics^24^ and if this is the case, then at the point of symptom onset in iCAA we would expect amyloid-β seeds to have maximally propagated throughout the CNS (i.e., that the patient is in the plateau phase); indeed we might reasonably assume that following spinal procedures, amyloid-β seeds must have reached the brain, otherwise we would not observe CAA. Notably, there was no difference in the latency (i.e., time between exposure to first presentation) in patients with spinal vs brain surgery. One explanation for the higher ICH recurrence after supratentorial procedures might be regional factors; tissue injury sustained at the time of the original operation might increase susceptibility to hemorrhage in the presence of amyloid-β pathology at a later stage; alternatively, factors relating to the index ICH might act as a trigger for further localized hemorrhagic events, akin to the temporal and spatial clustering of ICH observed in sporadic CAA.^14,15^ Another prion concept of potential relevance is that of “strains,” where multiple different prion conformations exist in a given host, with adaptation and mutation of these strains under different biologic selection pressures.^23^ If this concept also applies to amyloid-β assemblies—a hypothesis for which there is preliminary evidence^25,26^—then amyloid-β seeds propagating from relatively distant sites (such as the spine) may be subject to different selection pressures, with eventual selection and dominance of strains that are less likely to cause intracranial hemorrhage. Experimental interrogation of these potential amyloid-β strains and their structures, for example using high-resolution cryogenic electron microscopy, may shed light on the biologic basis for our observations.

Aside from intracranial hemorrhagic events, we also found a high rate of cognitive impairment in patients with iCAA. This finding could be directly related to injury after a high number of ICH,^27^ detected with high sensitivity by the detailed and frequent workup of patients in this study. Alternatively, the underlying vasculopathy of CAA can affect cognition independent of ICH,^28^ and recent data indicate that Alzheimer disease can also be acquired iatrogenically^29,30^ and thus might also make a contribution to cognitive impairment in this setting.

The principal strength of this study lies in the inclusion of—to the best of our knowledge—the largest cohort of iCAA with the longest available follow-up. Review of patients was mostly consistent across participating centers (typically with yearly outpatient follow-ups).

This study has several limitations. First, there is an inherent risk of selection bias. The cases described here are from large specialist tertiary centers with CAA expertise, so identification and referral to these centers might introduce selection bias towards more severe or unusual cases of CAA. Patients with most severe manifestations of CAA (e.g., large volume ICH), might not have survived the index event and therefore might have died before a diagnosis of iCAA could be made, leading to in advertent exclusion of these cases. Second, cognitive assessment was not standardized across centers; evaluations were based on locally used clinical tools (MoCA, more differentiated neuropsychological tests). Third, most patients fulfilled the criteria for “possible” iCAA, which are inherently less specific than the criteria for “probable” iCAA. Although we did not observe differences in prognosis between these groups, diagnostic misclassification cannot be fully excluded. Fourth, we did not have comprehensive structural imaging markers for all patients, limiting our ability to comment on CAA severity or individual radiologic markers that might be associated with increased ICH recurrence. Finally, the rarity of iCAA with identification of most cases in the past few years limited sample size, statistical power, and follow-up duration.

In conclusion, we further established a high rate of ICH in patients with iCAA after symptomatic presentation (about 20% per year). This risk appears to depend both on the type of exposure and first clinical event, with patients with brain surgery and an ICH as first symptomatic event having the highest rates of ICH recurrence. Larger studies with further extended follow-up, possibly based on the ongoing international iatrogenic CAA registry, will play an important role in clarifying and extending our findings.

## Glossary

cSAH: convexity subarachnoid hemorrhage
iCAA: iatrogenic cerebral amyloid angiopathy
ICH: intracerebral hemorrhage
MoCA: Montreal Cognitive Assessment
